# The effect of childhood exposure to secondhand smoke on urinary cotinine and 8 hydroxy-2-deoxyguanosine levels: A cross-sectional study

**DOI:** 10.18332/tid/240349

**Published:** 2026-10-23

**Authors:** Asuman Akkaya Fırat, İlkay Keskinel, Müzeyyen Eryılmaz, Haluk Kılıç, Şirin Güven

**Affiliations:** 1 Health Sciences University, Fatih Sultan Mehmet Training and Research Hospital, Medical Biochemistry Laboratory, Istanbul, Turkey; 2 Faculty of Medicine, Chest Diseases Department, Istanbul Okan University, Istanbul, Turkey; 3 Department of Internal Medicine, Health Sciences University Fatih Sultan Mehmet Training and Research Hospital, Istanbul, Turkey; 4 Department of Pediatrics, Health Sciences University, Sancaktepe Şehit Prof. Dr. İlhan Varank Training and Research Hospital, Istanbul, Turkey

**Keywords:** passive exposure to tobacco products, urinary cotinine, 8-OHdG, thirdhand smoke, secondhand smoke

## Abstract

**INTRODUCTION:**

The health consequences of exposure to tobacco smoke during childhood can be more severe than those associated with exposure in adulthood. Exposure to secondhand smoke is also a significant issue for children, who constitute a vulnerable group. While urinary cotinine measurements serve as metabolic indicators of nicotine exposure, measurements of 8-hydroxy-2-deoxyguanosine (8-OHdG) reflect DNA damage. This study evaluates the results of urine analysis obtained from healthy children, classified according to household smoking status.

**METHODS:**

In this cross-sectional study, urinary cotinine and 8-OHdG levels of a total of 97 children living in smoke-free homes, where only one adult smoked, and more than one adult used tobacco, were analyzed as a ratio to creatinine using an enzyme-linked immunosorbent assay kit. Families of children visiting the pediatric outpatient clinic for routine examinations were categorized based on their smoking habits. Additionally, a questionnaire using the Beliefs About Third-Hand Smoke (BATHS) scale was administered to these parents to evaluate their attitudes and beliefs regarding smoking.

**RESULTS:**

Urinary cotinine/creatinine levels were determined as 35.6 ± 5.17 pg/mg creatinine in the non-smokers (NS) group, 57.35 ± 4.84 pg/mg creatinine in the one person smoked (OPS) group, and 89.2 ± 5.3 pg/mg creatinine in the multiple people smoked (MPS) group. A statistically significant difference was found among all three groups (p<0.001). Urinary 8-OHdG levels differed significantly between the NS group and the other groups. However, no statistically significant difference was found when comparing the smoking OPS and MPS groups (p>0.05). Furthermore, we found a negative correlation between these two parameters and the BATHS score.

**CONCLUSIONS:**

Our study has shown that biomarker levels considered indicators of childhood secondhand smoke exposure may be associated with factors such as the number of smokers in the home. Furthermore, the findings suggest that the attitudes and beliefs of parents and household members regarding smoking may also be linked to the intensity of exposure.

## INTRODUCTION

It is noted that tobacco and tobacco products are the most widely used and addictive substances worldwide^1^. Although the harmful effects of tobacco smoke have been proven, approximately 40% of children aged 8–11 years in the United States are exposed to it^2^. It has been reported that families living in poverty are less likely to replace household items contaminated by smoke, and that children from low-income households are exposed to tobacco more frequently than children from high-income households^1^. Exposure to tobacco smoke at a young age has far more serious health consequences than exposure in adulthood. Research findings suggest that exposure to these adverse environmental factors during early life can lead to adaptive and functional changes during development, which may result in adverse outcomes in adulthood^1,3^. Nicotine in tobacco smoke is considered the most common cause of death and illness. Secondhand smoke exposure remains a significant public health issue, particularly among children. Passive smoking, or secondhand smoke (SHS) exposure, is when a person unknowingly inhales smoke from a burning cigarette. Thirdhand smoke (THS) refers to exposure to chemical residues left on surfaces after the use of tobacco products^4^. Even if a smoking area is ventilated, nicotine, lead, and carcinogens can cling to indoor surfaces for months. This poses an invisible yet serious threat to infants and children. Because their respiratory, immune, and nervous systems are still developing, children are much more severely affected by these toxic residues than adults^4,5^.

The cotinine biomarker is the gold standard for estimating nicotine exposure. Specific enzymes in the cytochrome P450 system in the liver convert most nicotine into cotinine. Cotinine, as the primary proximal metabolite of nicotine, has a half-life of approximately 15 hours and is widely recognized as an objective biomarker of exposure to cigarette smoke. It can be analyzed in blood, serum, saliva, and urine^6,7^. The combined effects of all substances in tobacco smoke increase oxidative stress (OS) and reduce antioxidant capacity, damaging lipids, proteins, and DNA in cell membranes^8^. Increased intracellular oxidative stress can damage membrane lipids, proteins, and DNA. This nucleoside indicates simultaneous damage to both the base and sugar components, which are known to contribute to the development of various diseases^9^. Increased intracellular oxidative stress can damage membrane lipids, proteins, and DNA. This effect results specifically from the formation of C5' radicals following the attack of hydroxyl radicals on 2'-deoxyribose sugar units, followed by cyclization with the C8 position of the purine base^8,9^. The presence of 8-deoxyguanosines (8-OHdG) in urine is considered a marker of DNA damage. This nucleoside indicates simultaneous damage to both the base and sugar components, which contributes to the development of various diseases^8,9^.

In practice, children’s exposure to secondhand smoke is typically assessed through caregiver or parent surveys. Since the questionnaires we administer alongside urine tests are more likely to be subject to bias, accuracy and sensitivity may be negatively affected^9,10^. This study aims to investigate the effects of environmental factors and parental attitudes regarding nicotine dependence during childhood by analyzing cotinine and 8-OH-guanosine levels in the urine of healthy children from both smoking and non-smoking families^10,11^.

## METHODS

### Study design and participants

This cross-sectional, observational, and controlled investigation enrolled healthy children aged 9–11 years who presented to the pediatric outpatient clinic for a routine pediatric examination. In this study, ‘healthy’ status was defined as having normal age-appropriate growth and development parameters, and the complete absence of any active acute infections or known chronic systemic diseases. Initially, 105 children were screened. After applying the selection criteria and excluding eight participants whose body mass index (BMI) fell outside the age-appropriate normal range, the final study cohort comprised 97 participants^12^. Following the routine medical examination, all consenting families completed a structured questionnaire (Table 1). Survey items were adapted from the Beliefs About Third-Hand Smoke (BATHS) scale, which was established through a comprehensive literature review by various universities^13^. Additionally, a specific item evaluating whether breathing air from a smoking room harms the health of infants and children was sourced directly from Winickoff-inclusion criteria^14^. The inclusion criteria were healthy children aged 9–11 years who presented to the pediatric outpatient clinic for a routine pediatric examination and whose parents agreed to complete the ethics committee informed consent form.

**Table 1:** Baseline demographic characteristics of the study population according to household smoking status, Türkiye, July 2024–August 2025 (N=97)

*Characteristics*	*NS (N=31)* *n (%)*	*OPS (N=34)* *n (%)*	*MPS (N=32)* *n (%)*	*p1*	*p2*	*p3*
**Age** (years)				0.54	0.58	0.72
9	10 (32.3)	12 (35.3)	10 (31.25)			
10	11 (35.5)	10 (29.4)	12 (37.5)			
11	10 (32.3)	12 (35.3)	10 (31.25)			
Mean ± SD	10.0 ± 1.6	9.0 ± 1.8	9.0 ± 2.1			
**Gender**						
Female^a^	16 (51.61)	17 (50)	16 (50)	0.85	0.77	0.54
Male^a^	15 (48.38)	17 (50)	16 (50)	0.73	0.31	0.68
**Household members**				0.73	0.61	0.59
≤5	17 (54.8)	16 (47.1)	15 (46.9)			
>5	14 (45.2)	18 (52.9)	17 (53.1)			
**Caregiver education level**				0.56	0.66	0.65
No high school	23 (74.2)	25 (73.53)	25 (78.2)			
High school	8 (25.8)	9 (26.47)	7 (21.8)			
**Home ownership**				0.63	0.72	0.81
Resident	25 (83.87)	27 (55.88)	24 (32.35)			
Owner	6 (16.12)	7 (44.11)	8 (65.62)			
**Employment**				0.59	0.61	0.75
Employed	27 (87.1)	29 (85.3)	26 (81.2)			
Not employed	4 (12.9)	5 (14.7)	6 (18.8)			
**BATHS score**				0.01*	0.01*	0.01*
<15	-	-	8 (25)			
15–30	3 (9.67)	11 (32.35)	9 (28.12)			
>30	28 (90.32)	23 (67.64)	15 (46.87)			

Data are row percentages.

a BMI (kg/m^2^) female: 13.8–15.9; male: 16.0–18.6.

* p<0.05; p1: NS-OPS, p2: NS-MSP, p3: OPS-MPS.

### Exclusion criteria

Eight children whose body mass index (BMI) fell outside the age-appropriate range were excluded, as this factor could potentially affect creatinine clearance. In accordance with the principles of voluntary participation, children whose parents declined to provide written informed consent were also excluded.

### Smoking classification

The cohort was classified based on household smoking habits: non-smokers (NS; n=31), one person smoking (OPS; n=34), and multiple people smoking (MPS; n=32). All families strongly emphasized that no individual smoked directly in the presence of the children. Furthermore, smokers reported that they exclusively smoked in separate areas outside the child’s room, such as on the balcony, and routinely ventilated the smoking area.

### Biochemical variables

Urine samples were collected from all participants to determine urinary cotinine and 8-hydroxydeoxyguanosine (8-OHdG) levels. The samples were centrifuged at 2000–3000 rpm for 20 minutes, and the supernatant was stored in small Eppendorf tubes at -80°C until analysis. Enzyme-linked immunosorbent assay (ELISA) procedures were performed in duplicate according to the manufacturer’s instructions. Cotinine, 8-OHdG, and creatinine levels were measured using commercial ELISA kits obtained from BT Bioassay Technology Laboratory (Cat. No. E2043Hu, EA0048Hu; Jiaxing Korain Biotech, Shanghai, China) and Cayman Chemical (Item No: 502330; Ann Arbor, MI, USA).

During the assay, urine samples were added to pre-coated microplate wells to allow cotinine binding. A biotinylated human cotinine antibody was then added to form a complex, followed by the addition of streptavidin-HRP. After incubation, unbound components were removed via washing. A substrate solution was added to initiate a colorimetric reaction proportional to cotinine concentration, which was then terminated with an acidic stop solution. All pipetting steps were executed manually, and incubations were maintained at 37°C. Absorbance was measured at 450 nm using a microplate reader. Standard curves were plotted using known concentrations. Final concentrations were expressed in pg/mL for cotinine, ng/mL for 8-OHdG, and μg/mL for creatinine. The measurement ranges, sensitivities, and intra-/inter-assay precisions were as follows: 0.5–80 pg/mL, 0.019 pg/mL, <10%, and <12% for cotinine; 7–1500 ng/mL, 3.25 ng/mL, <10%, and <12% for 8-OHdG; and 0.273–35 μg/mL, 0.270 μg/mL, <10%, and <12% for creatinine.

### Ethics

The Ethics Committee of S.B.Ü approved the study protocol. Sancaktepe Training and Research Hospital, in accordance with the Declaration of Helsinki (approval number: 180/12.6.2024). We provided detailed information to all participants, and they signed informed consent forms before inclusion in the study.

### Statistical analysis

We performed *a priori* power analysis to justify the target sample size before data collection. Power analysis indicated that, given our sample size, we had 90% power to detect an effect size of Cohen’s d=0.6 among the primary outcome measures at a significance level of α=0.05. We performed statistical analyses using SPSS version 22.0. Data are presented as frequencies and percentages, means and standard deviations, or medians and interquartile ranges (IQRs). We assessed normality using the Kolmogorov–Smirnov test. Univariate analysis was carried out using the Pearson chi-squared test for the categorical variables, independent samples t-test and Mann–Whitney U test for the normally distributed continuous variables, and the Kruskal-Wallis or one-way ANOVA test for the non-normally distributed continuous variables. A p<0.05 was considered significant. We evaluated the discriminative performance of the parameters, cutoff values, sensitivity, and specificity using ROC curve analysis and area under the curve (AUC) calculations.

## RESULTS

In our study, we found no significant differences in age or gender among families with no smokers (NS), one smoker (OPS), or multiple smokers (MPS) (Table 1) (p>0.05). We observed no statistically significant differences between groups in socioeconomic characteristics, including household details, caregiver training, home ownership, and employment status. We used a one-way ANOVA (or Kruskal-Wallis) test for numerical data such as age. In contrast, we used a chi-squared test of independence for categorical data.

Urinary cotinine/creatinine mean levels were determined as 35.57 pg/mg creatinine in the NS group, 57.35 ± 4.84 pg/mg creatinine in the OPS group, and 89.2 ± 5.3 pg/mg creatinine in the MPS group. A statistically significant difference was found between all three groups (p<0.001) (Table 2). Urinary 8-OHdG ng/mg creatinine values measured in the NS, OPS, and MPS groups were 165.80 ± 6.24, 317.70 ± 7.34, and 321.8 ± 5.3, respectively. There was a statistically significant difference among the three groups compared to the NS group (p<0.01). However, no significant difference was found between the OPS and MPS groups. BATHS score values measured in the NS, OPS, and MPS groups were 41.4, 35.3, and 17.9, respectively (p<0.01) (Table 2). To analyze these data, we used one-way ANOVA and the Kruskal-Wallis test to compare the three groups, and *post hoc* Bonferroni multiple-comparison tests for pairwise comparisons.

**Table 2:** Cotinine and 8-hydroxy-2-deoxyguanosine to creatinine ratios in urine samples from children in the NS, OPS, and MPS groups, Türkiye, July 2024–August 2025 (N=97)

	*NS (N=31)* *Mean ± SD*	*OPS (N=34)* *Mean ± SD*	*MPS (N=32)* *Mean ± SD*	*p1*	*p2*	*p3*
**Urinary cotinine pg/mg creatinine**	35.6 ± 5.17	57.35 ± 4.84	89.2 ± 5.3	0.01*	0.01*	0.01*
**Urinary 8-OHdG ng/mg creatinine**	165.80 ± 6.24	317.70 ± 7.34	321.8 ± 5.3	0.01*	0.01*	0.08

* p<0.05. p1: NS-OPS, p2: NS-MPS, p3: OPS-MPS.

Positive correlations were observed between urinary cotinine/creatinine concentration and 8-OHdG/creatinine concentration (p<0.01, r=0.690). Negative correlations were found between the BATHS scale and both urinary cotinine/creatinine concentration and 8-OHdG/creatinine concentration; the corresponding p and r values were (p<0.01, r= -0.803; p<0.01, r= -0.621), respectively (Table 3). We performed Pearson correlation analysis for these calculations.

**Table 3:** Correlation analyses between urinary cotinine and 8-OHdG and BATHS score in all cases, Türkiye, July 2024–August 2025 (N=97)

	*Urinary cotinine pg/mg creatinine*		*Urinary 8-OHdG ng/mg creatinine*		*BATHS score*	
	*p*	*r*	*p*	*r*	*p*	*r*
**Urinary cotinine pg/mg creatinine**	-	-	0.001*	0.690	0.01*	-0.803
**Urinary 8-OHdG ng/mg creatinine**	0.001*	0.690	-	-	0.01*	-0.621
**BATHS score**	0.01*	-0.803	0.01*	-0.621	-	-

* p<0.05.

We evaluated the discriminative performance of the parameters, cutoff values, sensitivity, and specificity using ROC curve analysis and area under the curve (AUC) calculations. The threshold value for cotinine/creatinine in urine was calculated as 49.23 pg/mL, with an area under the curve (AUC) of 0.93 (95% CI: 0.88–0.97, p<0.001), 87.2% sensitivity, and 84.5% specificity. The threshold value for Urinary 8-OHdG was calculated as 274.71 ng/mL, with an AUC of 0.92 (95% CI: 0.86–0.98, p<0.01), 84.6% sensitivity, and 82.8% specificity.

## DISCUSSION

Our findings indicate that the level of passive smoking to which people are exposed is directly influenced not only by the number of smokers in the household but also by the family’s attitudes and beliefs regarding smoking. The acute harmful effects of tobacco smoke on cellular health have been observed in both animal models and cell culture studies and are becoming increasingly apparent, particularly in children. However, its long-term consequences on human health have not been sufficiently studied. Furthermore, the literature highlights that understanding of tobacco smoke and its potential harmful effects is inadequate among the general public^15,16^. Communities and families play a crucial role in protecting children, toddlers, and infants from the dangers of tobacco smoke. The percentage of those who believe that tobacco smoke is harmful has been shown to range from 42.4% to 91%^17,18^. For tobacco smoke, it would be beneficial to use a valid, standardized tool to gather as much information as possible on knowledge, beliefs, and dissemination of THS^19,20^.

Park et al.^19,20^ analyzed urinary cotinine levels in children, both in absolute terms and relative to creatinine, using a liquid chromatography–tandem mass spectrometry system; they showed a direct correlation between urinary cotinine concentrations and the intensity of parental smoking. Exposure to secondhand smoke at home due to parental smoking habits is supported. While avoiding smoking at home can reduce children’s exposure to tobacco, they emphasized the need to identify other routes of exposure to secondhand smoke and ensure that smoking bans at home are properly monitored and enforced^19,20^.

In their study measuring 8-OHdG levels in urine using immunochemical methods in patients with type 2 diabetes due to increased oxidative stress load, Wu et al.^21^ reported that spontaneous 8-OHdG excretion relative to body mass index (BMI) is a significant indicator of glomerular filtration rate (GFR) and may be an important indicator in the early diagnosis of diabetes-related nephropathological changes. In our study, we applied a similar method. We analyzed 8-OHdG and cotinine levels, normalized to creatinine, in children in a similar age group with no significant differences in BMI^21,22^. In our study, we found no significant difference in the 8-OHdG/creatinine ratio between households with a single smoker and those with multiple smokers (p>0.05). This finding may reflect a ‘saturation effect’ (plateau effect) regarding passive smoke exposure; the presence of even a single smoker in the home might be sufficient to reach the observed level. Furthermore, the accumulation of smoke residues (‘third-hand smoke’) on indoor surfaces, creating a continuous basal exposure alongside exposures outside the home (outdoor, traffic, etc.) and other sources of oxidative stress, may have masked any potential difference attributable to the number of smokers in the household. As environmental measurements were not conducted, further studies are needed to elucidate this^23^. When evaluating the children in our study group regarding physiological and anatomical characteristics, we observed no significant respiratory or metabolic differences associated with puberty. In this age group, basal respiratory rates, minute ventilation volumes (adjusted for body surface area and BMI), and GFR values are quite similar between boys and girls^24,25^. Therefore, the amount of cotinine absorbed and subsequently metabolized by the lungs was expected to be similar in all children exposed to tobacco smoke at the same emission level. However, likely due to the extent of nicotine exposure, cotinine levels showed statistically significant differences across all three groups.

Our findings confirm that exposure levels to tobacco smoke (ETS) and nicotine are dose-dependent in households with multiple smokers. However, despite this marked increase in exposure, we did not observe a significant difference between groups in total 8-OHdG/creatinine ratios, which serve as an indicator of DNA oxidation. This paradoxical situation can be explained in two ways within the context of the relationship between secondhand smoke exposure and oxidative stress. Even the smoke load generated by a single smoker can induce an adaptive response. Consequently, although nicotine intake rises with an increase in the number of smokers, the magnitude of the compensatory response remains unchanged^25,26^. Alternatively, adaptive changes in endogenous antioxidants in response to chronic smoke exposure may have helped maintain a steady level of net oxidative damage despite the increased toxic load^27,28^. Given the delicate nature of homeostasis in children, they may demonstrate the capacity to develop an adaptive response that keeps net oxidative damage under control despite increased smoke exposure – whether stemming from a single individual or involving the chronic, high toxic load found in households with multiple smokers. These findings underscore the utility and necessity of using a valid, standardized tool to gather as much data as possible on knowledge and beliefs about tobacco smoke^29-31^.

It has been shown that the proportion of individuals who believe tobacco smoke is harmful ranges from 42.4% to 91%^17,18^. Regarding tobacco smoke, it would be beneficial to employ a valid and standardized tool to gather as much information as possible-drawing on data concerning knowledge, beliefs, and the spread of smoke^18^. Our study supports the view that the criteria used in the BATHS assessment serve as an effective indicator regarding awareness of the short- and long-term effects of smoking and their practical relevance. A higher level of awareness concerning the harmful effects of smoking was observed in the group with higher BATHS scores. While criteria 1, 2, 3, and 7 of the scale provide insight into short-term effects and the dangers of passive smoking (secondhand smoke), items 4, 5, 6, and 9 allow for the assessment of long-term effects. In particular, it was concluded that more compelling information is needed on long-term effects^14^. These results indicate that educational materials concerning exposure can raise awareness about passive smoking and that such materials should include more detailed information on the persistence of effects and environmental hazards.

In our study, no family members in the NS and OPS groups had a BATHS score below 15. However, all children had cotinine/creatinine levels in their urine samples exceeding 30 ng/mg, which is the threshold for thirdhand smoke (THS). This suggests that the children may be exposed to nicotine both indoors and possibly outdoors, indicating that the BATHS score does not provide a sufficient level of awareness to reduce the risk for individuals in the community. Although the BATHS scoring system appears to lack adequate protective measures, the severity of the effects decreased as the scores increased (for example, scores between 35 and 45). In contrast, lower scores (such as those between 15 and 30) were associated with the most intense effects.

Increased tobacco consumption in the home may pose a risk to children. This situation indicates that tobacco control strategies need to move beyond the mere emphasis on ‘no smoking at home’ and that more effective public health policies aimed at reducing the total burden of cigarette smoke in the home environment must be developed. Developing laboratory methods that offer high specificity, sensitivity, and reproducibility for the simultaneous analysis of urinary cotinine and 8-OHdG could prove beneficial. The study highlights the utility of these biomarkers in assessing exposure to cigarette smoke and the associated health effects of oxidative stress; their use in large-scale biomonitoring and public health surveillance programs should be supported.

### Limitations

The exclusion of children whose parents did not complete the ethical consent form or refused to sign the informed consent form may have introduced an unavoidable bias; families participating in the study might systematically differ from those who declined to participate or provided incomplete responses regarding smoking habits, health awareness, and the level of care shown to their children. This spontaneous situation may need to be overcome through homogenized, pre-designed, and larger cohorts. Our study was conducted on a relatively small group using a single urine sample and the ELISA method. Conducting this study in larger cohorts with repeated analyses of serum, saliva, skin, hair, and exhaled air samples, and using more sensitive techniques such as gas chromatography, would enhance its scientific validity.

## CONCLUSIONS

This study aimed to assess the intensity of exposure based on household smoking habits by measuring cotinine – a widely used urinary metabolite of nicotine – and 8-OHdG, a marker of cellular damage, as indicators of passive exposure to tobacco. Additionally, a questionnaire based on BATHS criteria was administered to evaluate the household’s attitudes and beliefs regarding smoking. Measurements using the BATHS scoring system yielded results consistent with the number of smokers and exposure levels. The use of this scale suggests that raising awareness about passive smoking exposure could be beneficial; as people become aware of the causes of children’s passive exposure to tobacco smoke, they are expected to be more willing to comply with rules aimed at preventing it.

## Supplementary Material



## Data Availability

Individual participant data that underlie the results in the article, after deidentification (text, tables, and appendices), will be made available from the corresponding author upon request. Data will be made immediately available to anyone who wishes to access the data, for any purpose, following publication. Data will be available indefinitely.
