# Supplementary material for: The effect of childhood exposure to secondhand smoke on urinary cotinine and 8 hydroxy-2-deoxyguanosine levels: A cross-sectional study
Source: Tob Induc Dis. 2026 Oct 23;24:10.18332/tid/240349. doi: 10.18332/tid/240349 (PMC13693463; doi:10.18332/tid/240349)
Supplement: Supplementary file 1 [file TID-24-194-s1.pdf]

Tobacco Induced Diseases

Supplementary file

© 2026 Akkaya Fırat A. et al.

DOI:
10.18332/tid/240349

The content has been provided by the author(s) and has not been reviewed, verified, or endorsed by European Publishing. It may not have undergone peer review. The views, opinions, and recommendations expressed are solely those of the author(s) and do not necessarily reflect the position of European Publishing. European Publishing accepts no responsibility or liability for any consequences arising from the use of, or reliance on, this content.

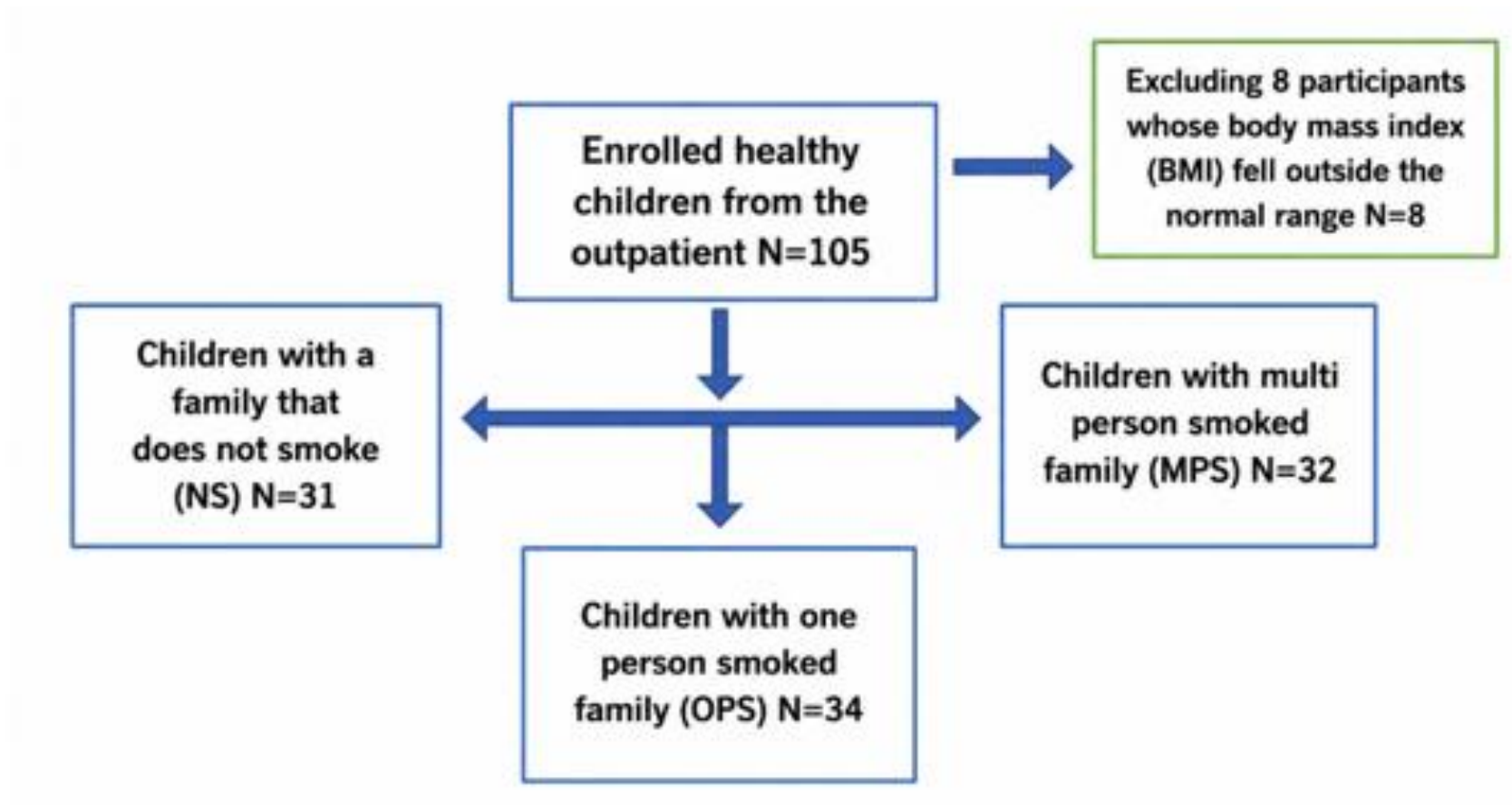

Figure1. Flowchart of the recruitment process

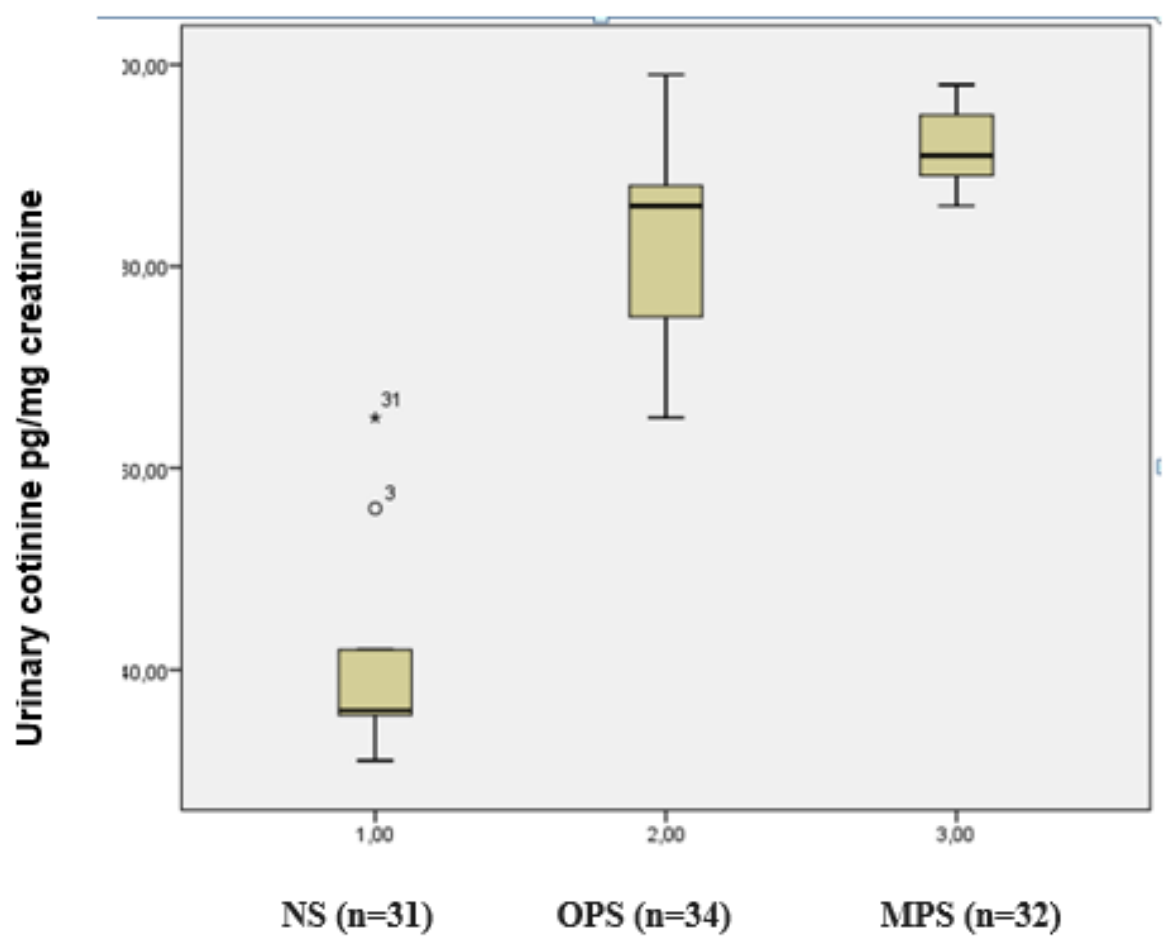

Figure 2. Cotinine-to-creatinine ratios in urine samples from children in the NS, OPS, and MPS groups (n=97)

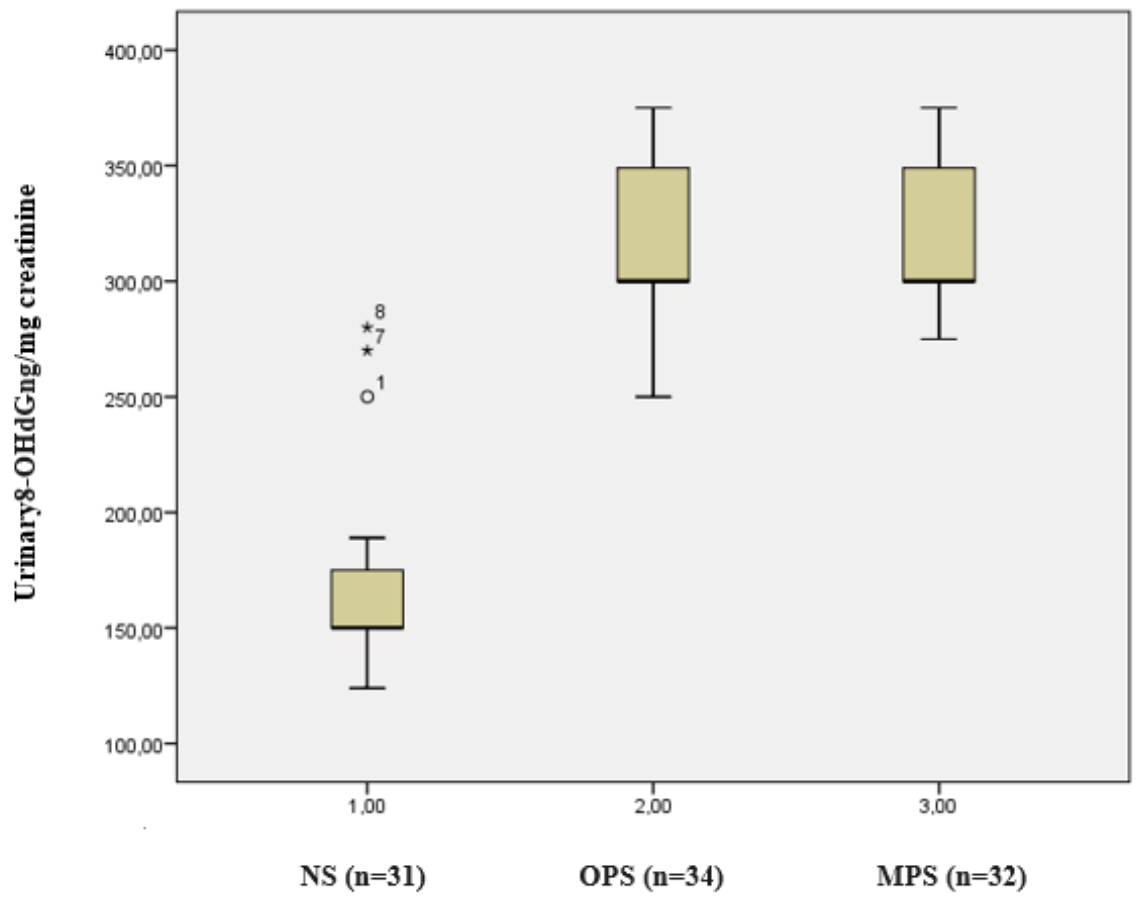

Figure 3. 8-OHdG -to-creatinine ratios in urine samples from children in the NS, OPS, and MPS groups (n=97)

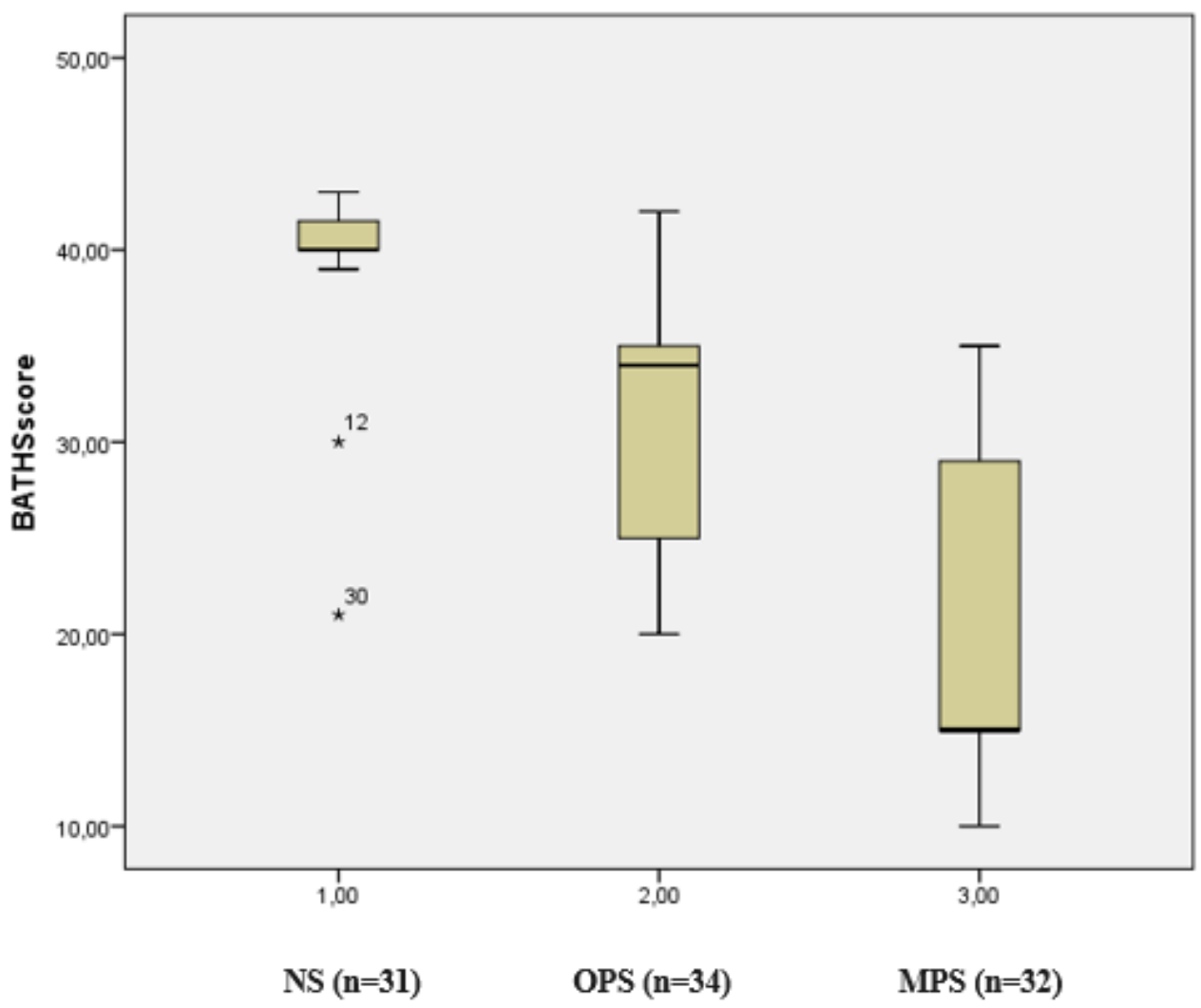

Figure 4. BATHS score distributions of the families of children in the NS, OPS, and MPS groups (n=97)

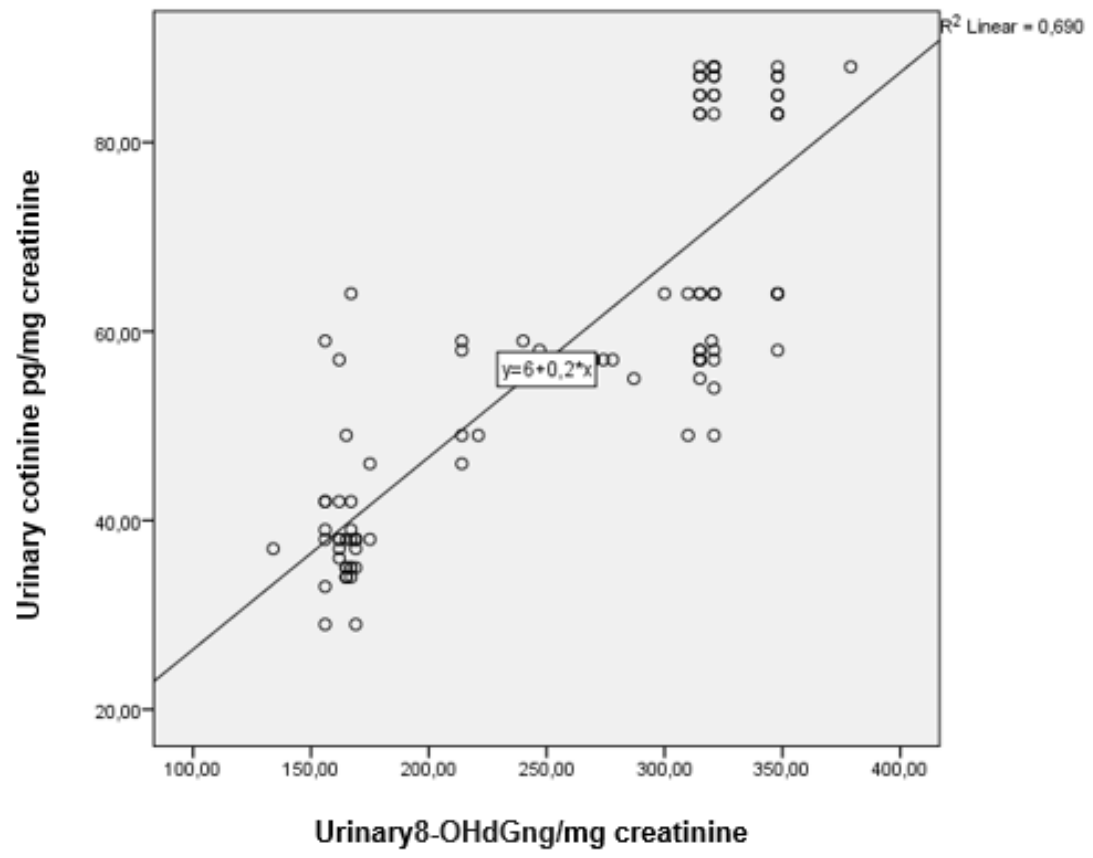

Figure 5. 8-OHdG to cotinin/ creatinine ratios in urine samples from children in the NS, OPS, and MPS groups.(n=97)

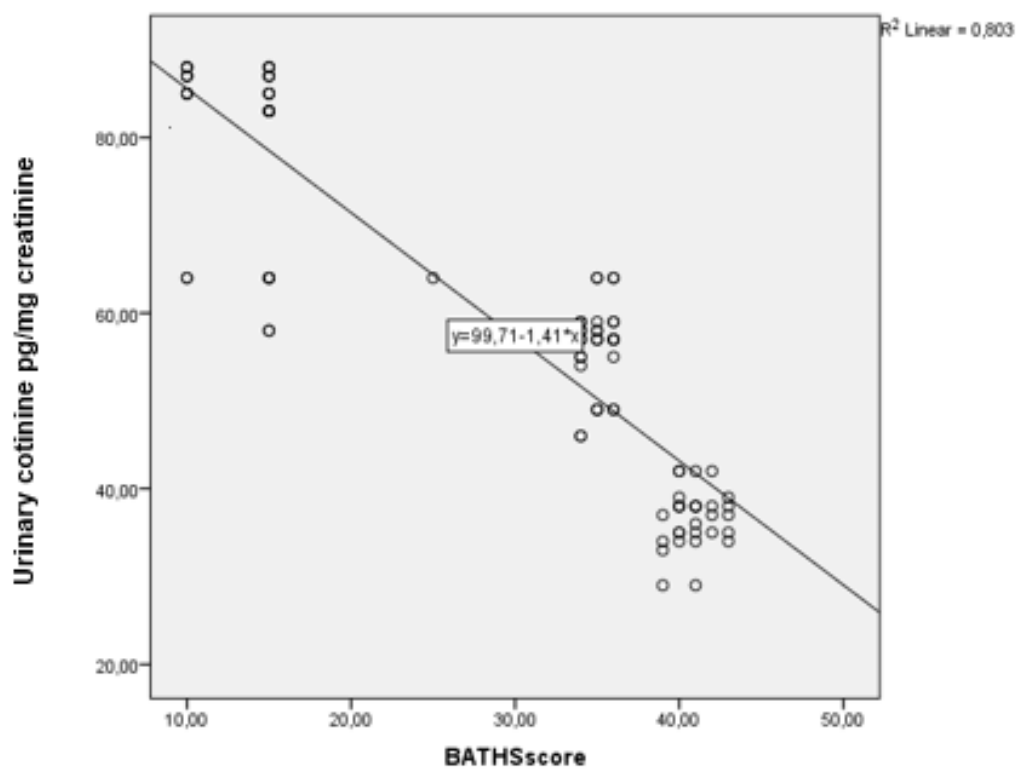

Figure 6. The relationship between the urinary cotinine/creatinine ratio and the BATHS score. n=97)

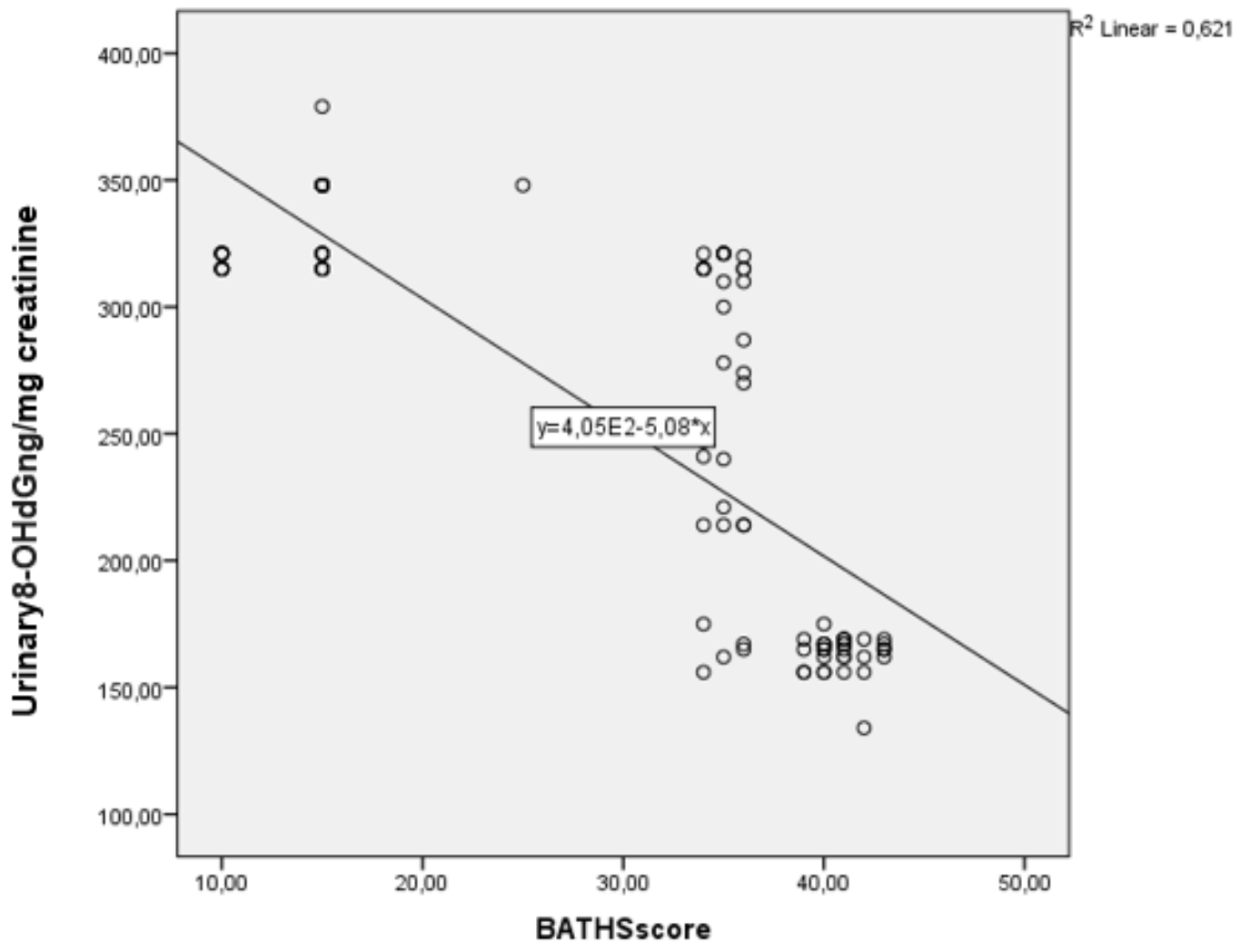

Figure 7. The relationship between the urinary 8-OHdG to/creatinine ratio and the BATHS score. (n=97)

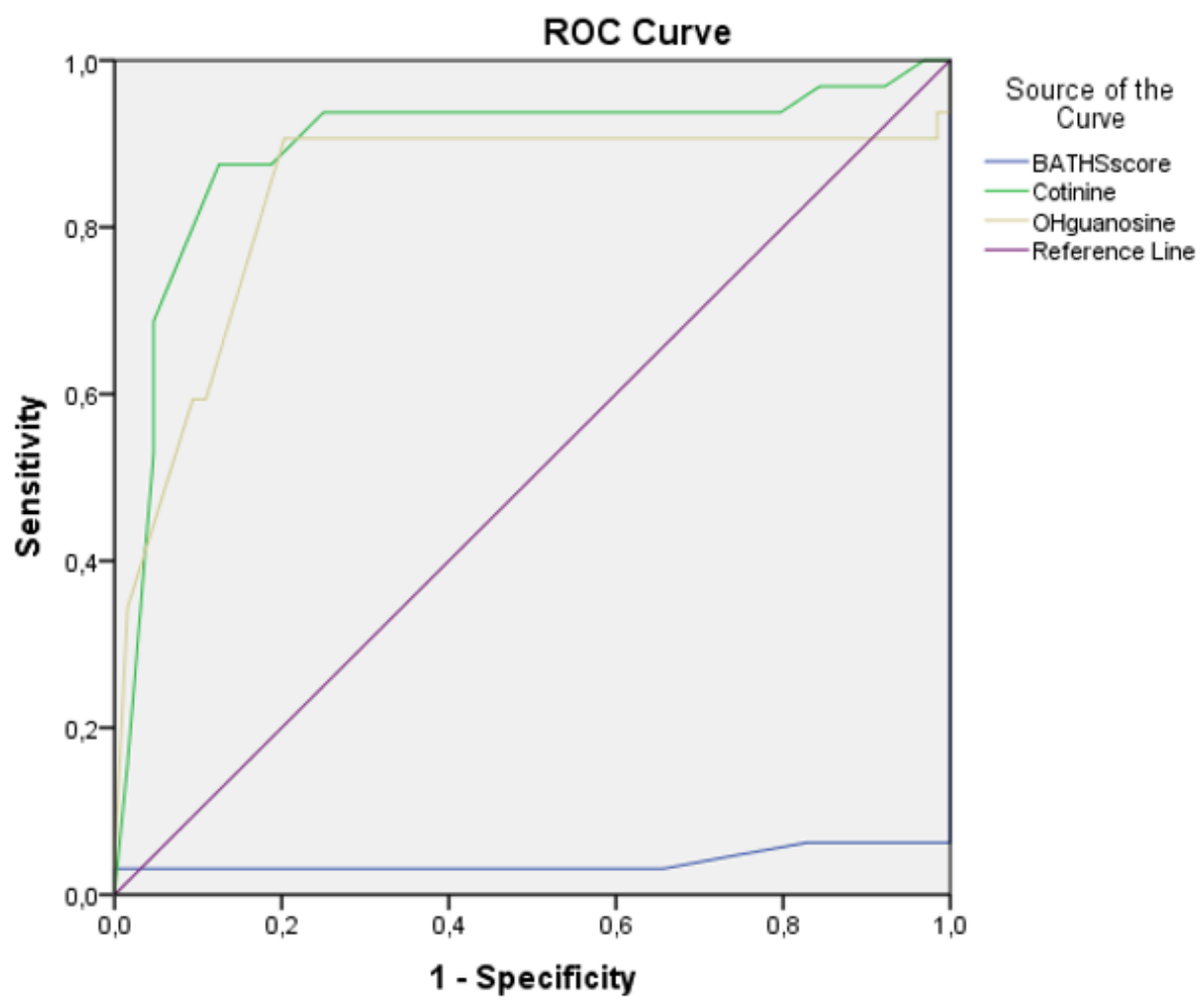

Figure 8. The ROC curve analysis of urinary cotinine/creatinine, 8-OHdG/creatinine, and BATHS score for predicting

Original BATHS scale

The next questions will ask for your opinions on the effects of smoking inside your home.	***Strongly disagree***	***Disagree***	***Not sure***	***Agree***	***Strongly Agree***
Breathing air in a room today where people smoked yesterday can harm the health of infants and children.	1	2	3	4	5
Breathing air in a room today where people smoked yesterday can harm the health of adults.	1	2	3	4	5
Particles in rooms where people smoked yesterday can cause cancer.	1	2	3	4	5
Smoke particles can remain in a room for days.	1	2	3	4	5
Smoke particles can remain in a room for weeks.	1	2	3	4	5
Smoke particles get absorbed into furniture and walls.	1	2	3	4	5
After smoking a cigarette, smoke particles on skin, hair, and clothing can be passed onto others.	1		3	4	5
After touching surfaces where cigarette smoke has settled, particles can enter the body through the skin.	1	2	3	4	5
Opening windows or using air conditioners does not eliminate all smoke particles in a room.	1	2	3	4	5
